# Country-specific costs of implementing the WHO FCTC tobacco control policies and potential financing sources

**DOI:** 10.1371/journal.pone.0204903

**Published:** 2018-10-03

**Authors:** Ce Shang, Amit Yadav, Michal Stoklosa, Anna Kontsevaya, Fabian B. Lewis, Adrian Pana, Irene Reyes

**Affiliations:** 1 Oklahoma Tobacco Research Center, Stephenson Cancer Center and Department of Pediatrics, University of Oklahoma Health Sciences Center, Oklahoma City, OK, United States of America; 2 Public Health Law and Advocacy, HRIDAY, New Delhi, India; 3 American Cancer Society, Atlanta, GA, United States of America; 4 National Research Center for Preventive Medicine, Moscow, Russia; 5 Ministry of Finance and the Public Service, Kingston, Jamaica; 6 Center for Health Outcomes and Evaluation, Sectorul, Romania; 7 Health Justice, Quezon City, the Philippines; University of Texas Medical Branch at Galveston, UNITED STATES

## Abstract

**Background:**

One of the major obstacles to the full implementation of the World Health Organization (WHO) Framework Convention of Tobacco Control (FCTC) tobacco control measures is the lack of sustainable financing resources.

**Goal:**

To update and simulate country-specific indicators that are highly relevant to the costs and financial resources of the treaty policy implementation. We also developed an Excel tool for simulation and assessed the aggregated-level indicators by the 2016 World Bank income groups.

**Approaches:**

Using mostly 2016 data or 2014–15 data if 2016 one are not available, we updated five indicators relevant to the treaty implementation, which are the gap between current and desirable policy implementation, cigarette affordability, the costs of implementing best- buy tobacco control policies, the number of smoking-attributable deaths, and the simulated tax revenue resulting from a $1 tax increase. We also aggregated indicators and simulation results by the World Bank income groups, encompassing the five indicators and the reduction in smoking and in attributable deaths due to a hypothetical 1I$ tax increase. Finally, the policy implementation cost was compared with tax revenue and revenue increases.

**Findings:**

As of 2016, smoking remains one of the leading causes of premature deaths worldwide while the implementation of best-buy tobacco control policies was below the recommended levels. Meanwhile, there was room to further increase cigarette taxes and prices, as cigarettes remained affordable in many countries. The total costs of implementing best-buy policies in the next 15 years merely account for 8.3% of the 2016 excise tax revenue, indicating that a small proportion of annual tax revenue could fund the implementation of tobacco control policies recommended by the WHO FCTC.

**Conclusions:**

Increasing taxes could have a multiplier impact on curbing tobacco use through aiding the implementation of the WHO FCTC.

## Background

Tobacco kills nearly 8 million people every year, costs economies an equivalent of approximately 2% of world’s combined gross domestic product (GDP) and significantly contributes to most of the leading causes of death in the world.[1] Recognizing that tobacco control is a fundamental investment in human capital, an investment that leads not only to enormous public health gains, but also to long-term economic growth, Parties to the World Health Organization (WHO) Framework Convention of Tobacco Control (FCTC) acknowledged the need for a collective action to overcome obstacles that delay the tobacco control policy implementation.[2, 3] Despite this consensus, the level of the WHO FCTC implementation worldwide has only increased moderately in recent years.[4]

At the Sixth Session of the Conference of the Parties (COP6) in October 2014, the working group on ‘sustainable measures to strengthen implementation of the WHO FCTC’ reported that the lack of funding resources was the major obstacle to implementing tobacco control measures recommended by the WHO FCTC. The working group also stressed that the country-level information on the cost of the policy implementation and the cost of inaction (the current costs of the tobacco epidemic) is lacking.[5] For example, in the assessment of the WHO FCTC Parties’ needs, there are currently no questions on finances and resources. Therefore, one of the key recommendations of the working groups was to provide countries with estimates on the costs of the tobacco epidemic and the funds needed to implement tobacco control policies. This information regarding the gap between desired and existing funding would tremendously support country-level efforts and strengthen capacities to implement the WHO FCTC. The needs for advancing treaty’s policy implementation and funding these efforts were emphasized again at the COP7 in November 2016.[6]

The costs and financing sources for implementing WHO FCTC policies differ by countries’ income levels. According to the WHO, the implementation of the four best-buy tobacco control measures (tobacco tax increases, smoke-free policies, package warnings, and advertising bans) in all Low- and Middle-Income countries (LMICs) would cost a mere 11 US cents per person per year. The estimates include human resources and physical capital needed to plan, develop, implement, monitor, and enforce the policies.[7] Currently, international funding (including private donors, aid agencies, and multilateral organizations such as WHO) is only about one US cent per person per year on tobacco control in LMICs. One other cent comes from governments of LMICs that provide funding for tobacco control programs in their countries. The total investment in tobacco control in LMICs at only 2 cents per person per year is thereby insufficient compared to current needs.[8]

In response to the requests by the Working Group on Sustainable Measures, country-level information on both the funds needed and the cost of inaction for many countries exist. However, such information is scattered in multiple literature and platforms, yet to be combined, compared and updated. This study was aimed to compile and update, for each country and economy globally, the five important indicators regarding financing and implementing the WHO FCTC policies. The five indicators are the MPOWER scores, cigarette affordability, the cost estimates of implementing the WHO FCTC best-buy tobacco control measures, tax revenue if excise cigarette taxes were increased by one international dollar (I$), and number of smoking attributable death averted as a result of a I$ 1 excise tax increase. We also used the excise tax revenue simulation results and the cost estimates of the best-buy policy implementation to evaluate the ratio of increased tax revenue that can be used to fund the implementation of best-buy policies recommended by the WHO FCTC. As increasing tax is an important part of the WHO FCTC policies, this approach will shed light on whether the WHO FCTC can fund itself through implementing its taxation policies.

## Method, data sources and measures

### Overview

The selection of the five indicators was guided by both the cost-benefit analysis of policies and the economic analysis of sin taxes (excise taxes imposed on unhealthy behaviors). Excise taxes on addictive goods such as tobacco and alcohol have been considered as an important tax revenue source for governments since the publication of the *Wealth of Nations*. When the lack of funding sources becomes a major obstacle to further implementing the FCTC policies, it is important to show how increasing excise taxes can potentially be a funding source, along with the major costs and benefits of the FCTC policy implementation.

The five indicators were updated or calculated at the country level using 2016 information (or 2014–15 information when the 2016 one is not available), with details described below. Country-level specific indicators and simulation results under the scenario of a I$ 1 excise tax increase were reported using an Excel tool. Finally, we aggregated indicators and simulation results by the 2016 World Bank income groups, which classify countries into high-income countries (HICs), middle-income countries (MICs), and low-income countries (LICs).

### MPOWER scores

#### Method

Following Dubray et al (2015) and Ngo et al. (2017),[9, 10] in order to showing the progress of the WHO FCTC implementation, we constructed an MPOWER composite score by adding up the six MPOWER scores. Although this composite score does not evaluate the full scale of the WHO FCTC implementation or policies, it is nonetheless an important measure of the arguably most effective policies and their implementation progress.

#### Data source

WHO’s *reports on the global tobacco epidemic* published in 2017 provide “MPOWER” scores that measure the WHO FCTC policy implementation in six domains. Among these six scores, “M” score evaluates the level of monitoring tobacco use and prevention policies, “P” score measure laws or policies protecting people from tobacco smoke (e.g. smoke-free air laws), “O” score refers to offering help to quit tobacco use, “W” score refers to warning about the dangers of tobacco, “E” scores refers to enforcing bans on tobacco advertising, promotion and sponsorship, and “R” scores refers to raising taxes on tobacco. Each score has four or five levels, with a score of 1 representing a lack of data (missing data) and a score of 2–5 representing the weakest to strongest policies.

### Cigarette affordability

#### Data source

The 2016 measure of cigarette affordability, originally developed by Blecher and van Walbeek,[11, 12] was obtained from the *WHO’s report on global tobacco epidemic 2017*, Table 9.6.0 “Affordability of the most sold brand of cigarettes” (http://www.who.int/tobacco/global_report/2017/appendix-ix/en/). Specifically, cigarette affordability was measured using the percent of GDP per capita required to purchase 100 packs (2,000 sticks) of cigarettes. To calculate the affordability measure, the WHO authors used prices in local currency from different editions of the reports and GDP per capita estimates from the International Monetary Fund (IMF) World Economic Outlook Database. Additional information of country-specific adjustments can be found in the Excel Table published by the WHO.

### Simulated excise tax revenue, by an I$1 increase

#### Method

We used the method from Goodchild et al (2016) to simulate excise tax revenues if cigarette excise taxes were increased by I$1.00.).[13] To be more specific, we first estimated how price and consumption change as taxes increase and then used those intermediate estimates to generate tax revenue estimates after the tax increase.

As in Goodchild et al (2016),[13] we made the following assumptions: first, taxes are fully passed to retail prices. Using prices of the most popular brand of cigarette. Second, if taxes are levied in tiers or in *ad valorem* rates, the amounts of taxes on the most popular brand of cigarette were used as the baseline levels. Third, general consumption taxes (e.g., value added and sales taxes (VAT)), were assumed to rise proportionally on the basis of the retail or wholesale prices of the cigarettes. On the other hand, other kinds of taxes (e.g. import duties or surcharges) were fixed. Fourth, the producer price net of tax was assumed to increase in line with the global inflation rate. Thus, the new retail price in the model was the sum of the new industry price per pack after adjusting for inflation, the new excise amount per pack after adding an $I 1.00 increase, and the new VAT amount per pack.

Next, the price elasticity of cigarette sales (consumption) in high, middle, and low-income countries was taken as -0.3, -0.4, and -0.5, respectively. The number of packs of cigarette sold in response to the price increase (C*) was calculated as: C × (1+ ΔP ×ε_p) in which C is the baseline consumption, ΔP is the percentage change in the retail price and ε_p is the price elasticity of demand or cigarette sales. Then the total excise revenue under the simulated condition was calculated as the product of the excise per pack and the quantity of cigarette packs sold in the retail market, reflecting the increases in taxes and decreases in consumption if excises taxes were increased by $I1. This simulated excise tax revenue was compared with the baseline revenue without the hypothetical I$1 tax increase.

#### Data sources

The simulation model requires data of three parameters: 2016 cigarette retail prices and various taxes, cigarette consumption, and inflation rate. The detailed information on taxes and prices also came from the 2017 *WHO’s report on global tobacco epidemic*, which reports including price of a 20-cigarette pack of the most popular brand (in international dollars adjusted by purchasing power parity), specific excises, *ad valorem* excises, VAT, import duties, and other taxes. 2016 cigarette consumption was derived from per capita consumption reported by Tobacco Atlas. 2016 Inflation rate was obtained from the World Bank World Development Indicators.

### Simulated number of smoking-attributable deaths averted, by an I$1 increase

#### Method

The method for estimating the number of smoking-attributable deaths averted and the reduction in the number of daily cigarette smokers, as a result of an I$ 1 increase in cigarette excise taxes, was also based on Goodchild et al. (2016).[13] First, we calculated the number of daily cigarette smokers aged 15 or above, which was to multiply the population estimates for aged 15 or older by the adult cigarette smoking prevalence. Next, we assumed that the price elasticity for cigarette smoking prevalence is -0.15, -0.2, and -0.25 in high, middle, and low-income countries respectively. If the cigarette excise taxes were increased by I$1, the reduction in the number of daily cigarette smokers (S) in the 2016 adult population (aged 15 or above) was calculated as S × ΔP × *ϵ_p_* in which S is the baseline number of smokers, ΔP is the percentage change in retail prices, and *ϵ_p_* is the prevalence elasticity.

In order to estimate the number of smoking-attributable deaths averted due to the I$1 tax increase, we followed Goodchild et al. (2016) and assumed a relatively low risk of smoking-attributable death, which is 33% for conservative estimates. Another assumption made by this method is that 95% of smokers aged 15–29 who quit will avoid an early death. This percentage would drop to 75% for smokers aged 30 to 39, to 70% for those aged 40 to 49, to 50% for those aged 50 to 59, and to 10% for those aged 60 or above. This assumption leads to a global adjustment factor that is 74% based on the 2015 age profile of the world population. Finally, the number of smoking-attributable deaths averted was calculated using the following formula 33%×74%×ΔS (the reduction in the number of daily cigarette smokers).

#### Data sources

Population estimates for people aged 15 (in total and by age groups) or above came from the Institute for Health Metrics and Evaluation (IHME) and the United Nation Population Division. Cigarette smoking prevalence among adults came from a variety of sources, including the 2016 prevalence estimates by the Euromonitor International, and the most recent tobacco use/smoking prevalence estimates compiled by the WHO’s report on global tobacco epidemic 2017. For countries where the most recent tobacco use survey was conducted before 2011, the daily cigarette smoking prevalence estimates (defined as the percentage of population who smoke every day) came from the WHO global report on trend in tobacco smoking 2000–2025. In addition, some countries only reported estimates of cigarette smoking prevalence and tobacco smoking prevalence, rather than daily cigarette smoking prevalence. In those cases, we used the proportion of daily cigarette smoking among these more broadly defined smoking activities at the country level as an adjustment factor. This adjustment factor (proportion) can be derived using the country-level estimates reported in the WHO global report on trend in tobacco smoking 2000–2025. If the proportion adjustment factors were not available for the country, we then assumed that 85% of tobacco smokers were cigarette smokers, among whom 85% were smoking daily, which was chosen because most derived adjustment factors center around these numbers.

### The WHO FCTC implementation cost of best-but policies for the next 15 years

#### Method

In order to estimate the total cost of implementing the four best-buy policies recommended by the WHO FCTC for the next 15 years, we used the *NCDs Costing Tool* developed by the WHO (http://www.who.int/ncds/management/c_NCDs_costing_estimation_tool_user_manual.pdf). We updated the parameters required to impute the cost, which are enforcement scores of best buy factors, exchange rate (US$, 2015–2016), exchange rate (PPP, 2015), GDP per capita (US$, 2015), GDP per capita (PPP, 2015), population (2016), income group classification (2016), gas/Ltr. (US$, 2016), population above the age 15 years (2015–2016), labor force (2014–2015), and the government health spending as a percentage of total health spending (2014).

#### Data sources

2016 population estimates were taken from the World Factbook Central Intelligence Agency (CIA). Exchange rates, GDP per capita, gasoline price, labor force estimates, and government spending as a percentage of total health spending were obtained from the World Bank database and represented recent estimates that we obtained from 2014 to 2016, as described above. Enforcement scores were updated using the WHO’s report on global tobacco epidemic (MPOWER dataset).

## Results

Country-specific estimates of the five indicators can be found in the Excel attachment (S1 File Tool), which can be used as a tool to simulate changes in retail cigarette prices, annual cigarette consumption, excise tax revenue, daily cigarette smoking prevalence and number of smoking-attributable deaths. The 2016 country-level information is given in the tool, with the modules showing all parameters used in the simulation. Moreover, this Excel tool also allows for juxtaposing important indicators regarding financing sources and costs, such as excise tax revenue and the implementation costs of four best-buy policies for the next 15 years. Although the cost estimation module is not directly built-in in the current Excel tool, it can be calculated using the already published cost estimation tool by the WHO.[14]

Overall, the requirement of inputs depends on the goal of usage. If the 2016 information is sufficient, the tool can be taken as given. In the future, users will be able to use updated documents such as the *WHO’s report on global tobacco epidemic and population estimates* to easily conduct simulation in retail cigarette prices and excise tax revenue based on the method in Goodchild et al, by coping and pasting future tax and price information to the tool. The users can also change fixed parameters such as price elasticity as needed. The updated cigarette consumption and daily smoking prevalence estimates may be obtained from the open data sources such as WHO reports and Tobacco Atlas.

Table 1 shows by income groups the five indicators that we consider are relevant to current WHO FCTC implementation and financing environment, which are the cigarette affordability, policy status measured by MPOWER scores, smoking-attributable deaths, cigarette excise tax revenue, and implementation cost of best-buy policies. The affordability indicators suggest that, on average, 18%, 6%, and 2% of 2016 GDP per capita were required to purchase 100 packs (2000 sticks) of cigarettes in LICs, MICs, and HICs, respectively. The average MPOWER scores for the three income groups were 17.5, 21 and 23, indicating that the level of policy implementation was below the recommended best-buy practices. The total number of smoking-attributable deaths among people who were alive in 2016 was estimated to be 9, 707, 230 in LICs; 262,509,500 in MICs; and 62,188,200 in HICs. In 2016, the estimated cigarette excise tax revenue totaled 2,683 million (I$) for LICs, 342,286 million (I$) for MICs, and 226,185 million (I$) for HICs. The combined policy implementation cost for the next 15 years is estimated to be 2,676 million (I$) for LICs, 30,812 million (I$) for MICs, and 13,971 million (I$) for HICs.

**Table 1:** Indicators related to tobacco control environment, policy implementation, costs, and financing, 2016.

Variable	LIC	MIC	HIC
Affordability (Average; N = 183)	18%	6%	2%
MPOWER score (Average; N = 183)	17.46	20.97	23
Smoking-attributable deaths in thousands (Total; N = 169)	9,707.23	262,509.5	62,188.20
Cigarette excise tax revenue, millions of I$ (N = 172)	2,682.80	342,286.19	226,185.19
Implementation cost for the next 15 years, millions of I$ (N = 136)	2,676.07	30,811.94	13,970.05

Note: Among 183 countries, 28 were LICs, 102 were MICs, and 53 were HICs.

Table 2 shows the aggregated simulation results if cigarette excise taxes in 2016 were increased by I$1 for LICs, MICs, and HICs, respectively. The results show that, if taxes were raised by $I1, total excise taxes would increase by 147% in LICs, 49% in MICs, and 26% in HICs. Retail prices would on average rise from I$3.06 to I$4.03 (32% increase) in LICs, from I$5.13 to I$6.1 (19% increase) in MICs, and from I$8.02 to I$8.99 (12% increase) in HICs. The annual total cigarette consumption would reduce by 25% in LICs, 13% in MICs, and 5% in HICs. The combined annual excise tax revenue would increase from 2,683 to 5,871 million I$ (119% increase) for LICs, from 335,402 to 481,996 million I$ (44% increase) for MICs, and from 226,185 to 274,760 million I$ (22% increase) for HICs. The average prevalence of daily cigarette smoking would be lowered by 14% in LICs, 6% in MICs, and 3% in HICs. The total number of adult daily smokers would drop by 3.6 million in LICs, 49.7 million in MICs, and 4.3 million in HICs, corresponding with a drop in the number of smoking attributable deaths by 0.8 million in LICs, 12 million in MICs, and 1 million in LICs.

**Table 2:** Simulation results due to an I$1 excise tax increase, 2016.

Variable	LIC	MIC	HIC
Excise, I$/20-cigarettes pack (N = 183)			
2016 baseline	0.68	2.04	3.84
After simulated excise increase	1.68	3.04	4.84
Change%	147.1	49	26
Retail Price, I$/20-cigarettes pack (N = 180)			
2016 baseline	3.06	5.13	8.02
After simulated excise increase	4.03	6.10	8.99
Change%	31.7	18.9	12.1
Annual consumption, millions of 2-stick pack (N = 172)			
2016 baseline	4,862	211,684	61,248
After simulated excise increase	3,631	183,551	58,370
Change%	-25.3	-13.3	-4.7
Annual excise tax revenue, millions of I$ (N = 172)			
2016 baseline	2,683	335,402	226,185
After simulated excise increase	5,871	481,966	274,760
Change%	118.8	43.7	21.5
Prevalence of daily cigarette smoking (N = 172)			
2016 baseline, % of adults 15+	8.1	17.5	20.0
After simulated excise increase, % of adults 15+	7.1	16.5	19.4
Change%	-14.1	-5.7	-3.3
No. of adult daily smokers (thousands) (N = 169)			
2016 baseline	29,416	789,351	188,449
After simulated excise increase	25,801	739,650	184,141
Change	-3,615	-49,701	-4,308
No. of smoking attributable deaths (thousands) (N = 169)			
2016 baseline prediction	9,707	260,486	62,189
Predication after simulated excise increase	8,825	248,349	61,136
Change	883	12,137	1,052

Table 3 shows the policy implementation cost per year as a share of annual excise tax revenue if the four best-buy policies will be fully implemented in 15 years, for the 131 countries where such estimates are available. The combined annual costs of implementing policies are estimated to be 178 millions of I$ for LICs, 2,053 millions of I$ for MICs, and 931 millions of I$ for HICs, respectively. The ratio suggests that 6.94% of the annual excise tax revenue in LICs is required to fund the implementation of best-buy policies in a 15-year period. For MICs and HICs, this ratio is 0.63% and 0.42%, respectively. If excise taxes per pack of 20 cigarettes were increased by $I1, the ratio would drop to 3.54% for LICs, to 0.43% for MICs, and to 0.35% for HICs. If the funds needed to implement best-buy policies are compared to tax revenue increases due to a 1I$ tax hike, they merely account for 7.2% of the increased tax revenue in LICs, 1.4% in MICs, and 2.0% in HICs.

**Table 3:** Implementation cost per year as a share of annual excise tax revenue, 131 countries (Percentage).

Variable	LIC	MIC	HIC
Implementation cost per year (millions of I$)	178.4	2,052.67	931.34
Proportion of annual tax revenue needed to implement best-buy policies (%)	6.94	0.63	0.42
Proportion of **simulated annual** tax revenue needed to implement best-buy policies (%)	3.54	0.43	0.35
Proportion of **simulated tax revenue increase** needed to implement best-buy policies (%)	7.22	1.4	2.0

## Discussion

The WHO FCTC is the first WHO treaty with 181 Parties worldwide as of 2017. However, the progress of implementing tobacco control policies recommended by the Treaty has been moderate.[4] The measures at COP Six and subsequently at COP Seven reported the lack of funding sources as working group on Sustainable a major barrier to fully implementing the WHO FCTC. In this study, we developed a tool to present country-specific indicators that are highly relevant to the costs and financing of the implementation, which can be used by policy makers and stakeholders to identify potential financing sources such as excise tax revenues. Specifically, the tool presents the gap between the current and the desirable status of imposing the best-buy policies, the number of smoking-attributable deaths, the policy implementation cost, tax revenue, and the room to further increase cigarette taxes and prices. Another important function of the tool is to simulate the consequences of excise tax increases, including reduction in smoking, increases in tax revenue, and the number of smoking-attributable deaths averted due to tax increases.

It is important to note that higher taxes/prices is one of the factors that contribute to the reduction of smoking prevalence and cigarette consumption. Other factors include the adoption of the WHO FCTC (ratification), developing and enforcing legislation and policies, and building capacity for monitoring the implementation. In this context, the tool that the study developed would be very useful in terms of financing the implementation of these non-taxation policies. The total impacts on smoking reduction and averted deaths through financing the implementation of a comprehensive policy package could be much greater than what were simulated in this study, which was focused on taxation.

This tool also allows for an assessment of aggregated indicators by the 2016 World Bank income groups. The results suggest that cigarettes were very affordable in 2016, especially in MICs and HICs. For example, only 6% of GDP per capita is required for consumers in MICs to purchase 100 packs of cigarettes. The implementation levels of the WHO FCTC policies were greater for higher income groups. Nonetheless, as of 2016, countries across income levels lagged behind in implementing best-buy tobacco control policies. Worldwide, the total number of smoking-attributable deaths among people who were alive in 2016 was estimated to be 334 million. The total 2016 excise cigarette tax revenue amounted to $I 571,154 million—a $I168,982 million increase from the 2014 tax revenue.[13] In contrast, the total cost of fully implementing best-buy policies in the next 15 years is estimated to be $I 47,458 million, which is about 8.3% the annual cigarette tax revenue.

The combined evidence highlights the fact that as of 2016 smoking remains one of the leading causes of premature deaths worldwide while the implementation of tobacco control policies was below the recommended levels. Meanwhile, there was room to further increase cigarette taxes and prices, since cigarettes remain affordable in many countries. Compared to the excise tax revenue, the cost of policy implementation is much smaller, indicating that a small proportion of the existing tax revenue or tax revenue increases could fund the full implementation of the four best-buy policies recommended by the Treaty.

Simulation results illustrate that increasing excise taxes is a win-win strategy in reducing smoking and related harmful consequences while increasing tax revenue, particularly in low- and middle- income countries. The comparison of Treaty implementation cost per year with tax revenue and revenue increases further suggest that higher taxes could be a win-win strategy if a small portion of tax revenues is used as financial sources to fund the implementation of tobacco control policies. We estimated that only 0.42% of the annual excise tax revenue is needed to fund the implementation of four best-buy policies in the next 15 years in HICs. This number is higher for MICs and LICs, but nevertheless is a small share of a single year’s tax revenue. Furthermore, if excise cigarette taxes were increased by $I1, merely 1.4% of the revenue increase is needed per year to fund policy implementation in MICs. This number is 2–7% for LICs and HICs but remains small relative to the tax revenue increases.

Many countries and localities have dedicated part of tobacco tax revenues to tobacco control efforts or other health-related initiatives.[15] Successful models can be found in Thailand and the California state in the United States, where tobacco control programs and health promoting activities have resulted in significant public health benefits and savings in healthcare cost.[15, 16] Although earmarking tax revenues has been criticized for potentially discouraging governments from increasing taxes and for its inflexibility and inefficiency, mechanisms can be put into place to mitigate these shortcomings.[15] As this study shows, a modest proportion of one year’s excise tax revenue may be sufficient to fund the policy implementation in the next 15 years. Such arrangement does not necessarily require a long-term earmarking system that may be prone to budgeting issues and business cycles.

There are some limitations of this study. First, in order to simulate changes in excise tax revenue due to an I$1 tax increase, we made assumptions on price elasticity and a full pass-through of taxes to prices. Existing studies suggest that tax structures play an important role in how cigarette taxes are passed to prices, showing that complicated tax structures are less effective in raising prices.[17, 18] Therefore, models that take into account of tax structures may be more predictive of tax revenue changes as taxes increase (e.g., the WHO TaxSim Model). Despite this limitation, the simulation method used in this study has the advantage over the TaxSim model in its simplicity, as it demands fewer parameters. Second, as a crude measure of the WHO FCTC implementation, the MPOWER scores do not capture the full scale of the treaty or the nuances in the policy implementation. Third, we estimated the number of smoking-attributable deaths, which is only part of the cost that smoking incurs. Considering lost productivity and healthcare cost due to smoking, the public health benefits of tobacco control and the cost of inactions are much greater. Fourth, not all information used to construct cost estimate and smoking-attributable deaths came from 2016. Finally, although the current study provides simulation evidence on the impact of increasing taxes on reducing cigarettes consumption in general population, it does not account for different impacts among sub-populations, such as groups based on social class, education, gender, and mental health status, as these groups may have different smoking behaviors or respond differently to the increasing taxes of tobacco.

In sum, this study stresses the significant impact of increasing cigarette excise taxes on tobacco control. As the most effective tobacco control measure and one of the four best-buy policies, increasing taxes not only leads to public health benefits, but also increases tax revenues that can be used to finance the implementation of other tobacco control policies and efforts. Therefore, increasing taxes could have a multiplier impact on curbing tobacco use through these mechanisms and aid the implementation of the WHO FCTC. Future studies that evaluate the progress of treaty implementation due to tax increases and quantify the overall influences of taxation on public health are highly warranted.

## Supporting information

S1 File Tool (XLSX)
